# Brain microvasculature endothelial cell orientation on micropatterned hydrogels is affected by glucose level variations

**DOI:** 10.1038/s41598-021-99136-9

**Published:** 2021-10-04

**Authors:** Ana María Porras Hernández, Laurent Barbe, Hannah Pohlit, Maria Tenje, Maria Antfolk

**Affiliations:** 1 Department of Materials Science and Engineering, Science for Life Laboratory, Uppsala University, Uppsala, Sweden; 2 Department of Biomedical Engineering, Lund University, Lund, Sweden; 3 Biotech Research and Innovation Centre, University of Copenhagen, Copenhagen, Denmark

**Keywords:** Biomaterials - cells, Cell biology

## Abstract

This work reports on an effort to decipher the alignment of brain microvasculature endothelial cells to physical constrains generated via adhesion control on hydrogel surfaces and explore the corresponding responses upon glucose level variations emulating the hypo- and hyperglycaemic effects in diabetes. We prepared hydrogels of hyaluronic acid a natural biomaterial that does not naturally support endothelial cell adhesion, and specifically functionalised RGD peptides into lines using UV-mediated linkage. The width of the lines was varied from 10 to 100 µm. We evaluated cell alignment by measuring the nuclei, cell, and F-actin orientations, and the nuclei and cell eccentricity via immunofluorescent staining and image analysis. We found that the brain microvascular endothelial cells aligned and elongated to these physical constraints for all line widths. In addition, we also observed that varying the cell medium glucose levels affected the cell alignment along the patterns. We believe our results may provide a platform for further studies on the impact of altered glucose levels in cardiovascular disease.

## Introduction

Endothelial cells line blood vessels throughout the body, in different microenvironments ranging from large arteries to microvascular veins, and substantial differences have been observed comparing endothelial cells from different sources^1^. Brain microvascular endothelial cells constitute a vital part of the blood brain barrier with the role to provide a protective environment for the brain. These endothelial cells are part of the brain microvasculature that constitute a unique subset of non-fenestrated vessels, that allow them to control the transport of molecules between the brain and the rest of the body^2^. This control is achieved by various means, where the tight junction proteins binding the brain microvascular endothelial cells tightly together play a specifically important role as they hinder paracellular flux of harmful substrates to reach the brain during normal homeostasis^3^. Different disease states, among these diabetes, have been shown to affect the endothelial cell functions and thus might ultimately alter the barrier properties^4^.

Microfabrication techniques such as micropatterning of surfaces or microfluidics have been extensively utilized to study endothelial cells from various different regions of the body^1,5–7^. It is well-studied and demonstrated that cell adhesion can be spatially controlled by micropatterning cell adhesion peptides or proteins on otherwise inert surfaces^8^. Similarly, endothelial cells have been seen to respond to micropatterned lines by elongating and aligning with the line direction, organizing their nuclei and actin fibres in parallel with the micropatterns and adopting an atheroprotective phenotype^5–7,9–12^. Furthermore, it has been shown that alignment on micropatterned surfaces alone is enough to influence gene expression of e.g. inflammatory genes and that cell shape determines cellular function^13,14^. These studies have however mostly included endothelial cells from larger vessels e.g. aortic cells or umbilical vein endothelial cells whereas the corresponding effects on brain microvasculature endothelial cells have not been studied in such detail.

Some important differences between large vein endothelial cells and microvasculature endothelial cells have been reported, where e.g. human umbilical cord endothelial vein cells (HUVECs) have been observed to align with flow-induced shear stresses^15^, whereas brain microvascular endothelial cells neither align nor elongate, or transitions from cobblestone to spindle-like morphology under shear stress or in response to curvature^16–18^. These different responses occurred despite HUVECs and the brain microvascular cell line b.End3 display similar responses to shear stress in terms of connexin37 expression, highly expressed in the healthy atheroprotective phenotype^19^.

In light of these differences in response to shear stress and curvature, we wanted to investigate the response of brain microvascular endothelial cells on micropatterned lines. Here, we patterned arginyl-glycyl-aspartic acid (RGD) peptide lines on hyaluronic acid hydrogels. We show the response of b.End3 cells, a mouse brain microvascular endothelial cell line, on different line widths (10–100 µm). Furthermore, we study the alignment in response to altered glucose levels to simulate hypo- and hyperglycaemia or altered blood glucose levels associated with diabetes. As several studies report that altered glucose levels have an effect on endothelial cell alignment in response to fluid-induced shear stress^20–22^ we wanted to understand if this would also affect the alignment of the brain microvascular endothelial cells when cultured on micropatterned lines.

## Results and discussion

In this paper we have investigated how brain microvascular endothelial cells respond to micropatterns of various widths. In addition, we report the behaviour of these cells under various glucose concentrations, emulating different blood sugar levels associated with diabetes.

### Brain microvascular endothelial cells adhere to micropatterned lines on hyaluronic acid acrylamide hydrogels

**Figure 1:**
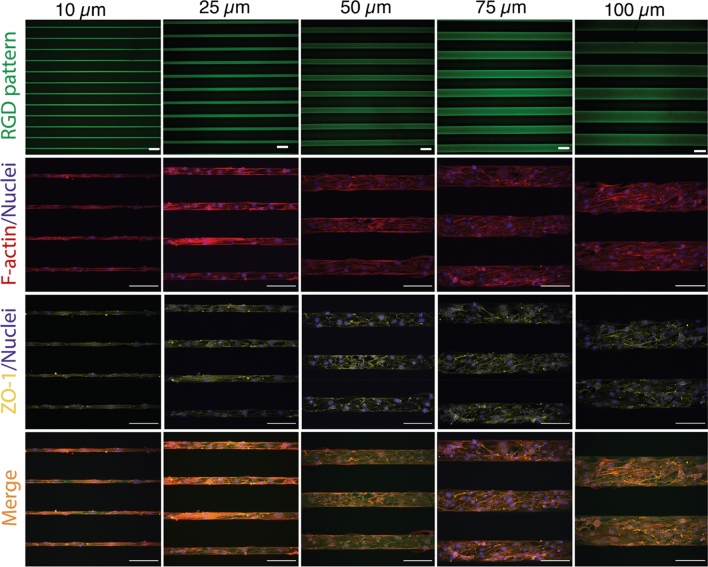
Brain microvascular endothelial cells adhere selectively to the RGD-patterned lines. First row: Lines of fluorescent 5-FAM-GCGYRGDSPG peptide patterned on HA-am hydrogels (green). Second row: b.End3 cells seeded on the lines stained for F-actin (red) and nucleus (blue). Third row: b.End3 cells seeded on the lines stained for zonula occludens-1 (ZO-1) (yellow) and nucleus (blue). Forth row: Merged images showing the cells on the micropatterned lines illustrating that the cells are adhering only to the peptide-patterned lines. Scale bars correspond to 100 µm in all images.

First, we wanted to study the behaviour of brain microvascular endothelial cells when seeded onto confined micropatterns of various widths, varying between 10–100 µm. To study the cells’ response to physical confinement on the micropatterned peptide lines, fluorescent RGD-peptide (5FAM-GCGYRGDSPG) lines were patterned on hyaluronic acid acrylamide (HA-am) hydrogels using a method previously described^23^.

b.End3 cells, a mouse brain microvascular cell line commonly used to study the blood brain barrier, were observed to adhere to lines of all line widths (Fig. 1). We could also confirm that the cells only adhered to the patterned surface and not to the bare HA-am hydrogel. Interestingly, here we found that the b.End3 cells could adhere even to the 10 µm wide lines, while in our previous paper, where we used the same b.End3 cell line, we observed that the cells would not adhere to 10 × 10 µm^2^ squares but required a minimum surface area of 25 × 25 µm^2^ for adhesion^23^. Extending one of the dimensions was apparently sufficient to provide enough adhesion sites for the cells.

### Peptide micropatterns direct nuclei and cell orientation in brain microvascular endothelial cells

**Figure 2:**
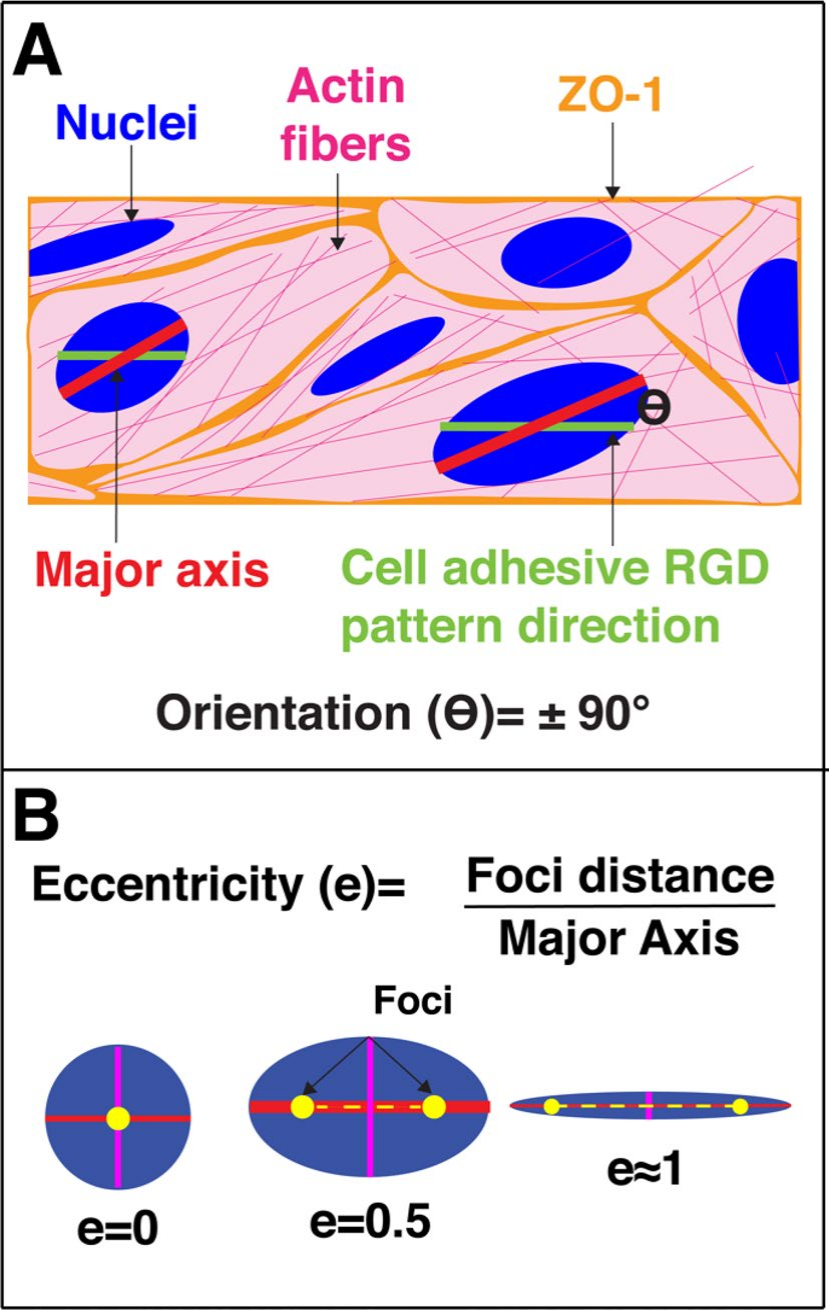
Schematic representing the data analysis. The nucleus, cell, or F-actin orientation with respect to the underlying peptide pattern and the nucleus or cell elongation was analyzed using Cell Profiler. (**A**) The cell, nuclei and F-actin were segmented from the images and fitted to an ellipse. The major and minor axis of the ellipse representation of the objects are identified. The orientation is determined by the angle between the major axis of the object and the underlying RGD pattern direction. (**B**) The nucleus or cell eccentricity indicates the elongation, where 1 is a line and 0 is a perfectly round circle.

Next, we investigated the extent of alignment the cells displayed on the different line widths looking at nuclei and cell orientations. The orientation of the nuclei or cells were measured with respect to the underlying RGD pattern direction. The orientation of the RGD pattern was set as 0° and the orientation of the nuclei or cells were defined as the angle between this RGD pattern and the cell or nucleus major elliptical axis (Fig. 2A). The nucleus or cell orientation is given in degrees, ranging from ± 90°. These experiments were performed with a glucose concentration of 25 mM, as recommended by the ATCC as initial culture conditions for b.End3 cells.

**Figure 3:**
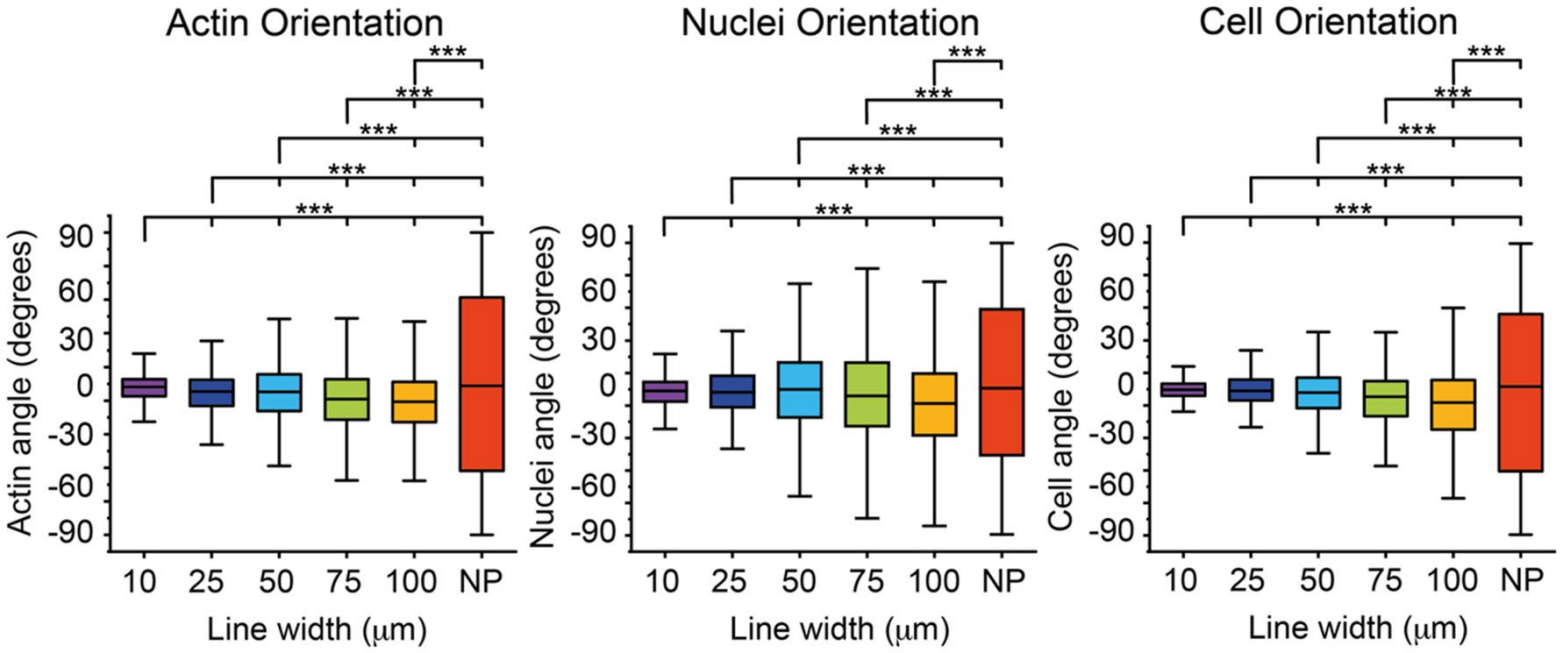
Brain microvascular endothelial cells align with the direction of the peptide pattern and alignment increases as the line width decreases. Box plots of actin orientation, nuclei orientation and cell orientation for the different line widths 10 µm, 25 µm, 50 µm, 75 µm, 100 µm, as well as a non-patterned (NP) control where the whole surface was covered with RGD. For the statistical analysis 400 nuclei and cells and 4000 actin fibres were used for each condition. The statistics included in figure 3 are summarized in Supplementary Tables S1, S2, S3, and *denotes *p* ≤ 0.05, **denotes *p* ≤ 0.01, and ***denotes *p* ≤ 0.001.

The nuclei and cell angle distributions on the different line widths are summarized in Fig. 3. Here, we observed that the brain microvascular endothelial cells do in fact orient themselves with the line direction, as the degree of both the nuclei and cells orientations on all lines (10–100 µm) differs from the random orientations observed on the non-patterned surface (denoted NP in Fig. 3) where the whole hydrogel was functionalized with RGD peptides. Previous observations have shown that brain microvascular endothelial cells do not orient themselves in response to shear stress or curvature^16–18^. This suggests that cell alignment response in brain microvascular endothelial cells on micropatterns are governed by pathways different from those responsible for responses to shear stress or curvature, as has also been observed previously in HUVECs^12^. Furthermore, the nuclei orientation angle decreased with decreasing line width, demonstrating an increased alignment of the cells. This is even more apparent when looking at the cell orientation. And indeed it has been previously shown that cell elongation and orientation is a key parameter of the nuclei deformation process^24^. Our results points to that a robust cell orientation event might precedes the nuclei orientation event, as seen by a clearer trend when it comes to the cell orientation as compared to the nuclei orientation.

On the contrary, nuclei and cells orientation on the non-pattern samples were widely distributed, which is as expected for a randomly oriented sample. These results show that in the absence of a line pattern of adhesion peptides, cells are randomly distributed on the surface and do not spontaneously arrange themselves without symmetry breaking cues.

### Peptide micropatterns induce directionality of F-actin orientation in brain microvascular endothelial cells

F-actin, as part of the intracellular cytoskeleton, plays a critical role in regulating endothelial cell alignment, and plays a major role in shaping and orienting the nucleus^25^ and an intermediary between the cell and nuclei orientation and shape^26^. The alignment of the F-actin fibres is often studied as a complement to the nuclei and cells orientation and has been reported to show a clear alignment trend as the pattern width decreases for several different cell types and in both two and three dimensions^27,28^. Thus, we set out to explore the orientation of the F-actin in response to the different line widths. The angle of each F-actin fiber in relation to the underlying RGD peptide pattern was defined as the F-actin orientation (Fig. 2A). The orientation of the RGD pattern was set as 0° and the F-actin orientation is given in degrees, ranging from ± 90°. The F-actin angle distributions as observed on different line widths are summarized in Fig. 3. We found that the endothelial cells displayed an aligned F-actin cytoskeleton on all line widths as compared to the non-patterned hydrogel (denoted NP in Fig. 3) which was completely functionalized with RGD peptides on the whole surface. In addition, we observed a higher degree of alignment on the smaller line widths than on the larger.

### Peptide micropatterns induce nuclei and cell shape change in brain microvascular endothelial cells

**Figure 4:**
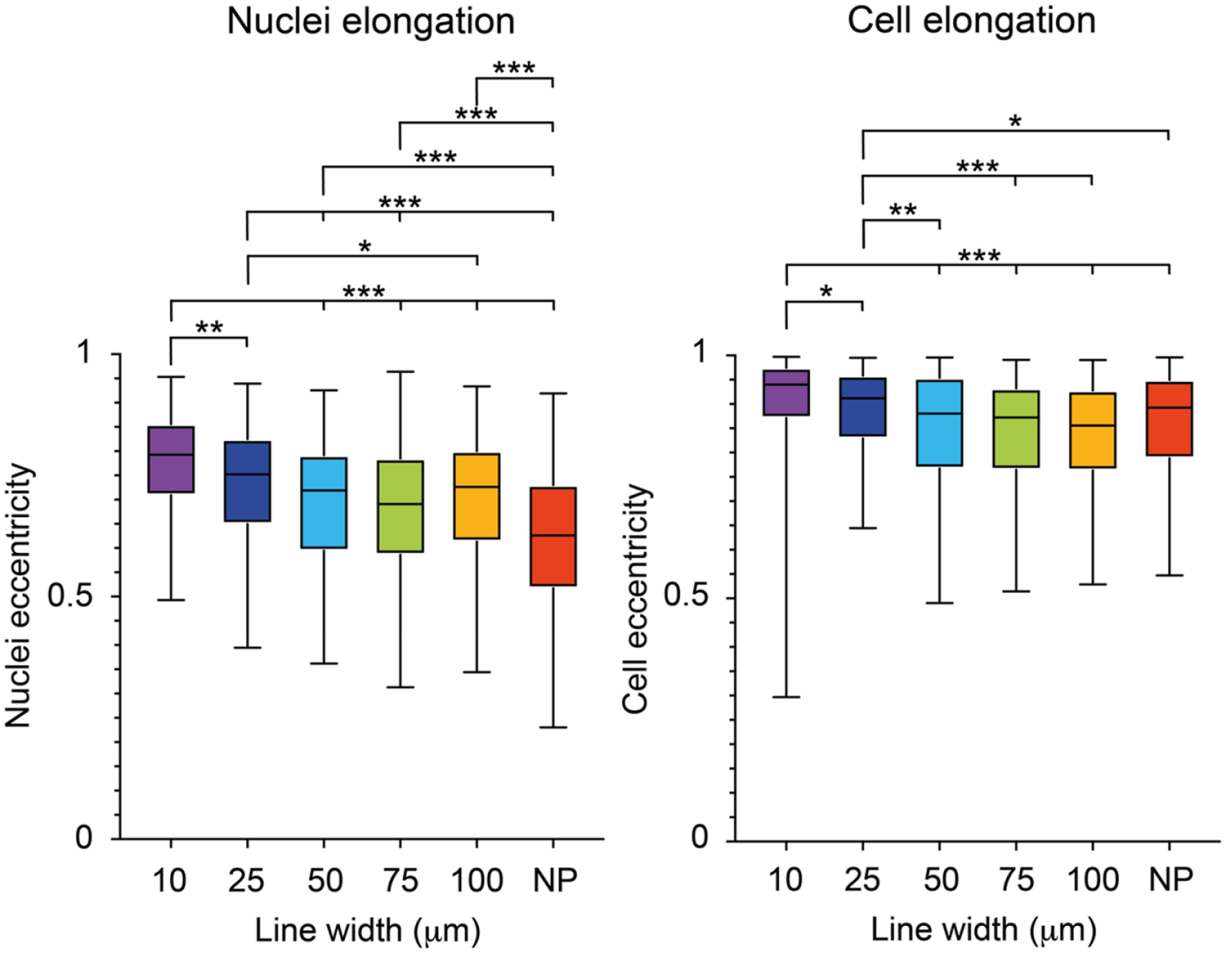
Nuclei and cell elongation of brain microvascular endothelial cells. Box plots of nuclei and cell elongation dependent on the line width (10, 25, 50, 75 and 100 µm and NP samples) where 0 denotes a round structure and 1 a line. For the statistical analysis 400 nuclei and cells were used for each condition. The statistics included in figure 4 are summarized in Supplementary Tables S4, S5, and *denotes *p* ≤ 0.05, **denotes *p* ≤ 0.01, and ***denotes *p* ≤ 0.001.

Next, we studied the nuclei and cell elongation determined by the nuclei or cell eccentricity (where 0 corresponds to a perfectly round shape and 1 to a line), as another measure to study cell-pattern alignment (Fig. 2B). Here, we observe a higher nuclei and cell eccentricity, meaning more elongated nuclei or cells, as the line width decreases showing that the cells are elongating to a higher degree along the narrower peptide line patterns, Fig. 4. Interestingly, for the cell elongation this effect starts to appear on the lines that are 25 µm wide or less whereas cells cultured on the 50–100 µm wide peptide lines showed a similar cell elongation as cells cultured on the non-patterned hydrogels**.** The effect is most strongly observed on the 10 µm wide peptide lines. This is in contrast with the nuclei elongation that is apparent, and statistically significant already at the 100 µm wide lines as compared to the non-patterned surface (NP).

The nuclei shape and orientation have been shown to be affected by the overall cell shape, mediated by the F-actin cytoskeleton^24^. Thus, we observe that a line width of 100 µm conforms the cell’s cytoskeleton enough to rearrange the nuclei orientation and shape, but not sufficiently to change the cell shape.

There is a clear relationship between the cell and the nuclei shape. Indeed, the shape of the nuclei is controlled by the cell shape through the actin filaments, especially the perinuclear actin cap that is a highly organised structure located above and around the nucleus^26^. This indicates that the nucleus become more and more oriented as the cell elongates and spreads along the micropatterned lines.

### Cells on the edge behave differently from cells in the centre of the lines

Of note, there has been previous reports of edge effects where the cell orientation and elongation has been observed to vary with the distance to the line edges. Culturing bovine aortic cells on 115 µm fibronectin-patterned lines, it was seen that the cell morphology varied depending on how far away the cell were from the line edges. The closer the cells were to the edges the more they displayed an elongated morphology^29^. Thus, we wanted to investigate if this effect was apparent in our experiments as well. Here, we observe a clear edge effect most prominent on the 100 µm lines, where the cells residing on the line edges are substantially more aligned compared to cells residing in the centre of the line (Supplementary Figs. S1 and S2). This is due to the fact that the cells on the line edges are more restricted in their movement as they are not able to orient themselves outside of the line. The cells in the line centre, on the other hand, are freer to orient themselves, both to the left and to the right, without any restrictions on one side.

Overall, in our study the brain microvascular endothelial cells responded to the micropatterned lines and displayed increased morphological alignment and elongation as line pattern width decreased. This is in line with previous studies using endothelial cells of different origins^5–7^. HUVECs were more aligned on 10 and 50 µm SVVYGLR peptide patterns on polyethylene terephthalate films compared to 100 µm and non-patterned substrates^11^. Similar alignment behaviour has been observed in bovine carotid cells grown on 15 or 30 µm wide collagen patterns in polystyrene culture dishes, whereas they displayed a loss of alignment when grown on 60 µm wide lines and non-patterned substrates^30^. However, it has also been shown that culturing HUVECs on 15 µm fibronectin patterns induced apoptosis after 12 h, while cells survived on 25 µm lines for 48 h^31^. Cell apoptosis on the narrower lines was not observed in our study. Clearly, it is challenging to compare these results as the experiments have been performed under different culture conditions e.g. different adhesion proteins, and cells were sourced from different species. It is not possible to draw any conclusions from these studies on where these different results stem from. However, it cannot be excluded that this can be a result of the difference in behaviour of endothelial cells depending on their origin within the body, and their inherent in vivo microenvironment, where e.g., the diameter of brain microvasculature is as small as 10 µm^32^, while larger blood vessels e.g. the aorta is several centimetres^33^.

### Hypo- or hyperglycosaemia affects both brain microvascular endothelial cell alignment and shape

Finally, we aimed to study the effects of hyper- or hypoglycaemia on brain microvascular endothelial cells and their resulting ability to respond to physical confinement under these different conditions. A common approach to emulate a diabetic effect in endothelial cells in vitro, is to elevate the glucose concentration of the cell culture medium to 25–35 mM in the hyperglycaemic case and lower it to 1 mM in the hypoglycaemic case. A concentration of 5.5 mM of glucose in the cell culture medium is normally used to represent healthy homeostasis^21,34,35^. Here, we seeded the cells on the 10 µm wide lines and varied the glucose concentrations, where 1 mM was used to simulate a low glucose level, 5.5 mM was used to simulate a normal glucose level, and 25 mM was used to simulate a high glucose level. The brain microvascular endothelial cells were kept in 25 mM glucose during routine culture, as recommended by the supplier, and the glucose concentration of the media was varied as the cells were seeded on the peptide-patterned hydrogels.

**Figure 5:**
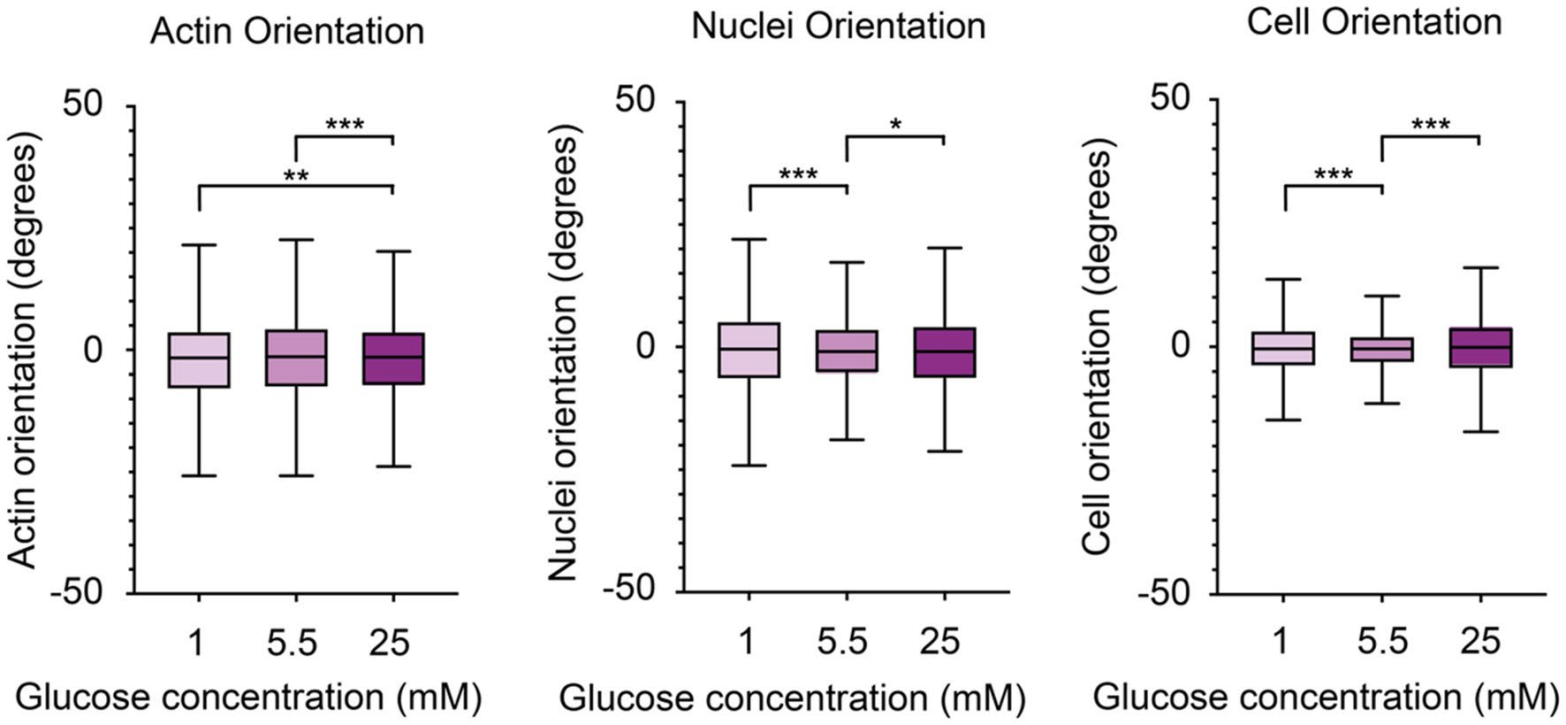
Brain microvascular endothelial cell orientation on 10 µm lines is affected by cell media glucose levels. Box plots of actin orientation, nuclei orientation and cell orientation for the different glucose conditions low (1 mM), normal (5.5 mM), and high (25 mM). For the statistical analysis 750 nuclei and cells and 7500 actin fibres were used for each condition. The statistics included in figure 5 are summarized in Supplementary Tables S6, S7, S8, and *denotes *p* ≤ 0.05, **denotes *p* ≤ 0.01, and ***denotes *p* ≤ 0.001.

Investigating the nuclei, cell, and F-actin orientations under the different glucose conditions, we observed noticeable differences. Both the cells and the nuclei were less oriented in cells cultured in either low or high glucose (1 mM or 25 mM), as compared to the cells culture under the normal glucose conditions (5.5 mM) (Fig. 5). Interestingly, the F-actin orientation displays an opposite trend where the F-actin of cells cultured in either low or high glucose was more oriented than that of the cells cultured under a normal glucose concentration. This trend becomes more apparent when looking at the variance differences than in the box plot. However, this difference was not statistically significant between the 1 mM and 5.5 mM conditions.

**Figure 6:**
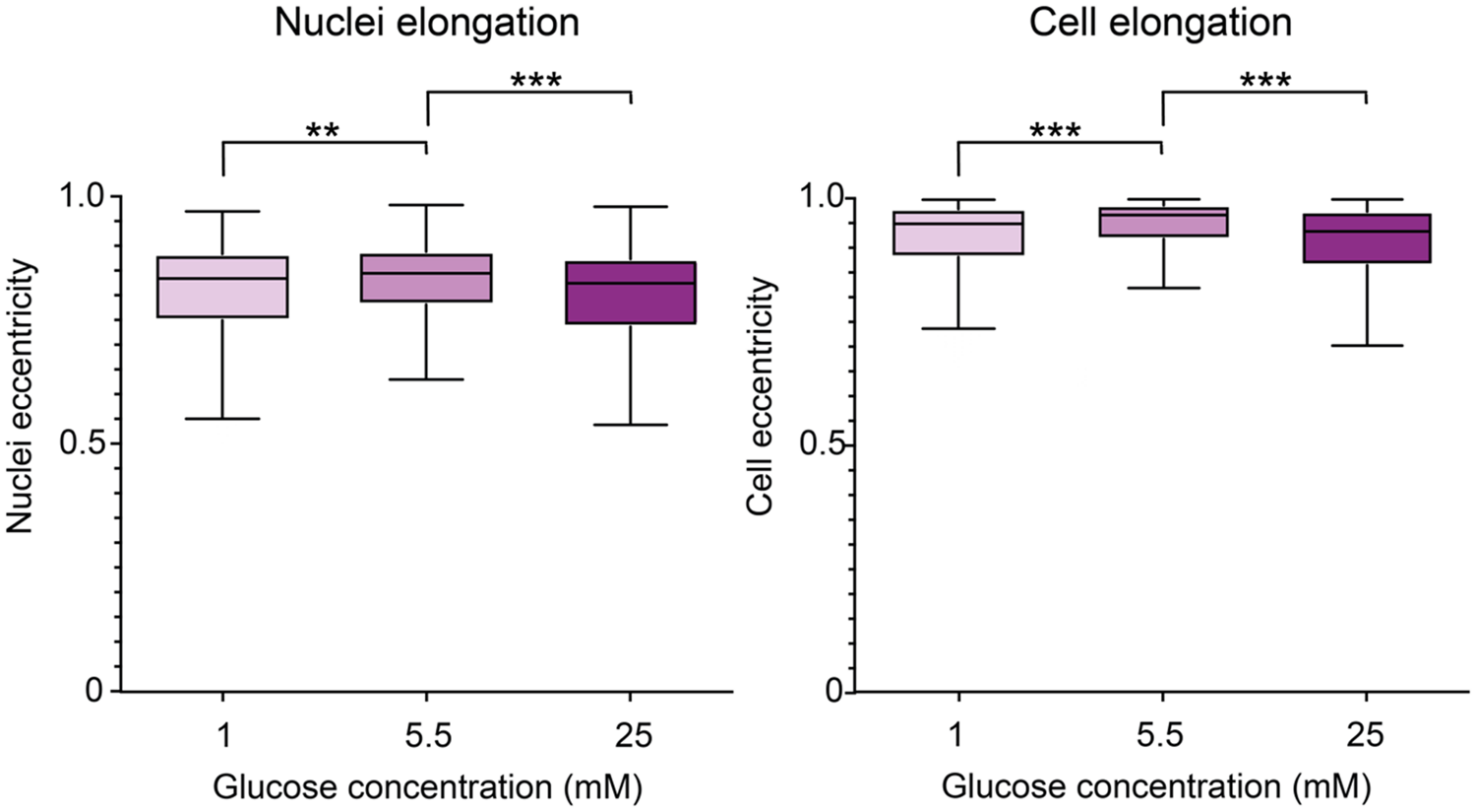
Nuclei and cell elongation of brain microvascular endothelial cells is affected by glucose levels. Box plots of nuclei and cell elongation dependent on the different glucose conditions low (1 mM), normal (5.5 mM), and high (25 mM), where 0 denotes a round structure and 1 a line. For the statistical analysis 750 nuclei and cells were used for each condition. The statistics included in figure 6 are summarized in Supplementary Tables S9, S10, and *denotes *p* ≤ 0.05, **denotes *p* ≤ 0.01, and ***denotes *p* ≤ 0.001.

Nuclear and cell elongation, as measured by the nuclei or cell eccentricity, was also affected by the different glucose conditions (Fig. 6). Both the cell body and the nucleus of the cells cultured under normal glucose conditions displayed a more elongated morphology, compared to both the cells cultured in the low and the high glucose medium. Together with the orientation differences above this indicate that both hypo- or hyperglycaemic conditions severely alters the cell function and its ability to regulate its shape and alignment.

Interestingly, here the F-actin orientation seems to defy the overall trends of both the cell and nucleus orientation and elongation. Both the nuclei and cell bodies are more oriented and elongated in cells culture under normal glucose conditions (5.5 mM) as opposed to cells cultured under either hypo- or hyperglycaemic conditions (1 mM or 25 mM glucose). However, the F-actin instead shows a trend of being more aligned under the extreme conditions than under normal glucose. Here, we also observed that the number of actin filaments per cell decreased under the extreme glucose conditions (Supplementary Fig. S3).

It has previously been established that the nuclei shape and orientation is dependent on the cell shape and regulated by the perinuclear actin cap, a dome-like actin structure that covers the top of the nucleus^26^. We therefore hypothesize that the cells cultured in the low or high glucose medium that display less oriented and elongated cell body do not possess the same degree of oriented perinuclear actin, which translated into a less oriented and elongated nucleus. Here, we also observed less F-actin fibers overall in these cells. Less perinuclear actin forming the actin cap would further explain why these cells cultured under the extreme conditions do not align and orient their nuclei to the same extent as the cells culture under normal glucose conditions.

In addition, there is a confirmed crosstalk between cell cytoskeleton and the metabolisms that is thought to reciprocally mediate each other^36^. The glycolytic enzyme aldolase is also involved in the formation of F-actin bundles by stabilizing parallel F-actin filaments, in favor of a less organized branched F-actin cytoskeleton. Higher availability of glucose promotes a higher level of glycolysis, which requires more aldolase to be involved in this cycle instead of stabilizing the F-actin^36^. This might mean that less aldolase is available to stabilize the formation of a more organized parallel F-actin cytoskeleton, and indeed we observed less F-actin in these cells. This in turn will result in a less oriented cell nuclei as the nuclei orientation is dependent on the highly organized perinuclear actin cap^37,38^. This supports our hypothesis that we observe less nuclei orientation during the high glucose condition because of a reduction in the organized parallel F-actin present in the perinucleus actin cap. In turn this could suggest that a lower availability of glucose resulting in a reduced glycolysis would, at least initially, mean that more aldolase would be available to stabilize and organize the F-actin cytoskeleton promoting a more oriented nucleus. However, this was not apparent in our results but might possibly be observable during an earlier experimental time point.

## Conclusions

In this study we have demonstrated that brain microvascular endothelial cells elongate and orient along micropatterned lines. This adds to the understanding of this highly specialized subgroup of endothelial cells, which have previously been shown to not respond with either elongation or alignment to shear stress or curvature. This demonstrates that elongation due to spatial confinement is governed by different mechanisms than response to shear forces or topography. Furthermore, we have shown that these brain microvascular endothelial cells are affected by alterations in the glucose levels, and that they respond differently both to low and high glucose in comparison to normal glucose conditions. Both lower and higher glucose levels promote a more atheroprone phenotype displaying less orientation along the micropatterns. We envision that this model could be used for further studies towards personalized cardiovascular treatments of diabetic patients.

## Materials and methods

### Synthesis of hyaluronic acid acrylamide derivative

Hyaluronic acid acrylamide (HA-am) was synthesized as previously reported^23,39^. Shortly, sodium hyaluronate (Lifecore Biomedical) was functionalized with acrylamide groups by reacting the carboxylic acid group of HA to an amino group of *N*-(2-aminoethyl) acrylamide linker (abcr GmbH). The final product was freeze-dried and kept at − 20 °C until use. The degree of modification was 14% as determined by ^1^H NMR (Supplementary Fig. S4).

### Patterned HA-am hydrogel preparation

Patterned hydrogels were prepared as previously described^23^. Briefly, Irgacure 2959 (Sigma-Aldrich) was dissolved in phosphate buffer to a final concentration of 0.4% (w/v). HA-am was dissolved in the Irgacure 2959 solution to a final concentration of 2.0% (w/v). The precursor solution was placed in a Si/SU8 mould and exposed to UV light (4.6 J/cm^2^) to initiate cross-linking of the hydrogel through a glass cover slip used as a lid to the Si/SU8 mould determining the hydrogel thickness. The resulting hydrogel film was then covered by a solution of 0.5 mM 5FAM-GCGYRGDSPG peptide (Innovagen AB) and exposed to UV light through a photomask (1.9 J/cm^2^) to define the chemically modified areas. The photomask consisted of patterns of straight lines with varying widths (10, 25, 50, 75 and 100 µm) at constant spacing (100 µm). Non-patterned control samples were prepared by exposing the HA-am hydrogel covered with peptide solution 5-FAM-GCGYRGDSPG through a photomask with an 8 mm × 8 mm opening (1.9 J/cm^2^). These samples are termed non-patterned (NP) hydrogels throughout the manuscript and were used as positive control samples to confirm cell adhesion onto the RGD peptide and negative control samples for the cell alignment study. Negative control samples for the peptide adhesion consisted of HA-Am hydrogels exposed to UV light (1.9 J/cm^2^) without the presence of RGD peptide solution.

### Cell culture and glucose conditions

Mouse brain microvascular endothelial cells b.End3 (purchased from ATCC) were cultured in Dulbecco’s Modified Eagle Medium Glutamax High Glucose (Gibco) supplemented with 10% fetal bovine serum (GE Healthcare Hyclone) and 1% Penicillin Streptomycin (Lonza), as recommended by the cell supplier, and maintained at 37 °C in 5% CO_2_. Cell media was renewed every two days. Cells were passaged when reached 80% confluency using TrypLE™ Express enzyme (Gibco) for dissociation. To study cell orientation related to physical confinement on the peptide-patterned lines, b.End3 cells, passage between 24 and 35, were seeded on the patterned and non-patterned control hydrogels in DMEM high glucose media at a density of 20,000 cells/cm^2^. The samples were kept in culture at 37 °C in 5% CO_2_ for 24 h until they were analysed. To investigate the effect of glucose levels on cell alignment, two additional media compositions were used; DMEM without glucose (Gibco) supplemented with 1 mM D-glucose (Sigma) and DMEM low glucose (Gibco) containing 5.5 mM D-glucose. To study the interplay of physical confinement and glucose level on cell alignment, b.End3 cells, passage between 24 and 35, were seeded on hydrogel samples with 10 µm wide line patterns and cultured in the media of varying glucose levels (1, 5.5 and 25 mM). Cells were seeded at a density of 20,000 cells/cm^2^ and the samples were kept in culture at 37 °C in 5% CO_2_ for 24 h, before fixed an analysed.

### Cell visualisation and alignment analysis

Cell alignment was evaluated after 24 h in culture. The cells were fixed in 2% paraformaldehyde in PBS. The samples were blocked for 2 h with a blocking buffer containing 3% bovine serum albumin (BSA) and 0.2% IGEPAL (Sigma) in PBS. Rabbit ZO-1 primary antibody (Invitrogen) was dissolved to 1:100 in 50% blocking buffer in PBS and incubated overnight at 4 °C. After, the samples were washed three times with Triton X-100 (0.3%). Goat anti-rabbit Alexa Fluor 568 (Invitrogen) was dissolved in 50% blocking buffer in PBS and incubated for 2 h at room temperature (RT). The samples were stained for F-actin using SPY620-Actin (Spirochrome) for 1 h at RT followed by nuclei staining with Hoechst 33,342 (2000×) for 20 min at RT. Cell images were acquired with a laser scanning confocal microscope (Leica SP8) equipped with a 25× water objective.

The cellular, nuclear and actin parameters were determined using Cell Profiler v 3.1.9^40^. Cell profiler segmented images of fluorescent nuclei, ZO-1 and F-actin. The orientation and elongation were based on the ellipse representation of the segmented object. The orientation is defined as the angle (θ) between the major axis of the ellipse and the direction of the RGD patterned lines, ranging from ± 90°, where 0° indicates the direction of the underlaying line pattern (Fig. 2A). The elongation was determined by the eccentricity of the fitted ellipse of the object. The eccentricity is defined as the ratio between the distance between the foci of the ellipse and the major axis. The eccentricity values range from 0 to 1, where 0 indicates a circle, and as the value approaches 1 indicates a more elongated morphology (Fig. 2B).

### Edge effect

To determine if there is an edge effect the 100 µm lines were used. The cell, nuclei and actin orientation, as well of the cell and nuclei eccentricity of cells located within 10 µm from the edge of the lines (top and bottom) were selected from the Cell Profiler output and compared against the values of the cells located in the center of the line, within ± 10 µm of the center of the line. A cell was deemed to be inside of either of these areas if its nucleus was inside or on the border line or the area. The cells that resided within these areas were analyzed as described above.

### Image collection and statistical analysis

For the line width experiment three gels per line width were analyzed. Four images were acquired per sample, resulting in 12 images per line width for image analysis. For statistical analysis 400 nuclei, 4000 actin fibers and 400 cells were randomly selected and analyzed in order to have the same number of cells and fibers from all different line widths.

For the glucose experiments three gels per glucose concentration were analyzed. This was repeated three times. Four images were acquired per sample, resulting in 12 images per glucose condition per experiment. For statistical analysis 750 nuclei, 7500 actin fibers and 750 cells were randomly selected and analyzed in order to have the same number of cells and fibers per condition.

As the alignment of the nuclei, cell and actin is always centered around 0 degrees, there is no difference between the means of these different populations. Instead, they differ because they have different variances. For the different alignment experiment we have, thus, statistically tested if there is a difference in variance between the different conditions using an Ansari-Bradley test adjusted for multiple comparisons.

For the nuclei and cell elongation there is a difference in means between the conditions and thus we have applied a more standard statistical approach to this data and compared differences of the means using a one-way ANOVA followed by a Tukey post hoc test. *P*-values less then 0.05 was considered statistically significant as follows where *denotes *p* < 0.05, **denotes *p* < 0.01, ***denotes *p* < 0.001.

## Supplementary Information

Supplementary Information.

## Data Availability

The datasets generated during and analyzed during the current study are available from the corresponding author on reasonable request.

## References

[1] Anderson DEJ, Hinds MT (2011). Endothelial cell micropatterning: methods, effects, and applications. Ann. Biomed. Eng..

[2] Wolff, A., Antfolk, M., Brodin, B. & Tenje, M. In vitro blood-brain barrier models: An overview of established models and new microfluidic approaches. *J. Pharm. Sci.* (2015) 10.1002/jps.24329 25630899

[3] Daneman R, Prat A (2015). The blood-brain barrier. Cold Spring Harb. Perspect. Biol..

[4] Haspula D (2019). Influence of a hyperglycemic microenvironment on a diabetic versus healthy rat vascular endothelium reveals distinguishable mechanistic and phenotypic responses. Front. Physiol..

[5] Dike LE (1999). Geometric control of switching between growth, apoptosis, and differentiation during angiogenesis using micropatterned substrates. Vitr. Cell. Dev. Biol. Anim..

[6] Gao D, Kumar G, Co C, Ho CC (2008). Formation of capillary tube-like structures on micropatterned biomaterials. Adv. Exp. Med. Biol..

[7] Leslie-Barbick JE, Shen C, Chen C, West JL (2010). Micron-scale spatially patterned, covalently immobilized vascular endothelial growth factor on hydrogels accelerates endothelial tubulogenesis and increases cellular angiogenic responses. Tissue Eng. Part A.

[8] Théry M (2010). Micropatterning as a tool to decipher cell morphogenesis and functions. J. Cell Sci..

[9] Kobayashi A (2007). In vitro formation of capillary networks using optical lithographic techniques. Biochem. Biophys. Res. Commun..

[10] Moon J, West J (2008). Vascularization of engineered tissues: Approaches to promote angiogenesis in biomaterials. Curr. Top. Med. Chem..

[11] Lei Y, Zouani OF, Rémy M, Ayela C, Durrieu M-C (2012). Geometrical microfeature cues for directing tubulogenesis of endothelial cells. PLoS ONE.

[12] Vartanian KB, Kirkpatrick SJ, Hanson SR, Hinds MT (2008). Endothelial cell cytoskeletal alignment independent of fluid shear stress on micropatterned surfaces. Biochem. Biophys. Res. Commun..

[13] Vartanian KB, Berny MA, McCarty OJT, Hanson SR, Hinds MT (2009). Cytoskeletal structure regulates endothelial cell immunogenicity independent of fluid shear stress. Am. J. Physiol. Physiol..

[14] Kidoaki S, Matsuda T (2007). Shape-engineered vascular endothelial cells: Nitric oxide production, cell elasticity, and actin cytoskeletal features. J. Biomed. Mater. Res. Part A.

[15] Kroon J (2017). Flow-induced endothelial cell alignment requires the RhoGEF Trio as a scaffold protein to polarize active Rac1 distribution. Mol. Biol. Cell.

[16] Reinitz A, DeStefano J, Ye M, Wong AD, Searson PC (2015). Human brain microvascular endothelial cells resist elongation due to shear stress. Microvasc. Res..

[17] DeStefano JG, Xu ZS, Williams AJ, Yimam N, Searson PC (2017). Effect of shear stress on iPSC-derived human brain microvascular endothelial cells (dhBMECs). Fluids Barriers CNS.

[18] Ye M (2014). Brain microvascular endothelial cells resist elongation due to curvature and shear stress. Sci. Rep..

[19] Pfenniger A (2012). Shear stress modulates the expression of the atheroprotective protein Cx37 in endothelial cells. J. Mol. Cell. Cardiol..

[20] Brower JB, Targovnik JH, Bowen BP, Caplan MR, Massia SP (2009). Elevated glucose impairs the endothelial cell response to shear stress. Cell. Mol. Bioeng..

[21] Kemeny SF, Figueroa DS, Clyne AM (2013). Hypo- and hyperglycemia impair endothelial cell actin alignment and nitric oxide synthase activation in response to shear stress. PLoS ONE.

[22] Hoesli CA (2018). Dynamics of endothelial cell responses to laminar shear stress on surfaces functionalized with fibronectin-derived peptides. ACS Biomater. Sci. Eng..

[23] Porras Hernández, A. M. *et al.* A simplified approach to control cell adherence on biologically derived in vitro cell culture scaffolds by direct UV-mediated RGD linkage. *J. Mater. Sci. Mater. Med.* **31**, (2020). 10.1007/s10856-020-06446-x PMC7560931 33057798

[24] Versaevel M, Grevesse T, Gabriele S (2012). Spatial coordination between cell and nuclear shape within micropatterned endothelial cells. Nat. Commun..

[25] Tamiello C, Buskermolen ABC, Baaijens FPT, Broers JLV, Bouten CVC (2016). Heading in the right direction: Understanding cellular orientation responses to complex biophysical environments. Cell. Mol. Bioeng..

[26] Khatau SB (2009). A perinuclear actin cap regulates nuclear shape. Proc. Natl. Acad. Sci. U. S. A..

[27] Ha M, Athirasala A, Tahayeri A, Menezes P, Bertassoni L (2020). Micropatterned hydrogels and cell alignment enhance the odontogenic potential of stem cells from apical papilla in vitro. Dent. Mater..

[28] Nikkhah M (2012). Directed endothelial cell morphogenesis in micropatterned gelatin methacrylate hydrogles. Biomaterials.

[29] Lin X, Helmke BP (2008). Micropatterned structural control suppresses mechanotaxis of endothelial cells. Biophys. J..

[30] Li S (2001). Effects of morphological patterning on endothelial cell migration. Biorheology.

[31] Wu C-C (2007). Directional shear flow and Rho activation prevent the endothelial cell apoptosis induced by micropatterned anisotropic geometry. Proc. Natl. Acad. Sci. U. S. A..

[32] Wong, A. D. *et al.* The blood-brain barrier: An engineering perspective. *Front. Neuroeng.* **6**, (2013). 10.3389/fneng.2013.00007 PMC3757302 24009582

[33] Hager A (2002). Diameters of the thoracic aorta throughout life as measured with helical computed tomography. J. Thorac. Cardiovasc. Surg..

[34] Wang Z, Chen G, Yu G, Liu C (2014). Pyrroloquinoline quinone protects mouse brain endothelial cells from high glucose-induced damage in vitro. Acta Pharmacol. Sin..

[35] Li W, Maloney RE, Aw TY (2015). High glucose, glucose fluctuation and carbonyl stress enhance brain microvascular endothelial barrier dysfunction: Implications for diabetic cerebral microvasculature. Redox Biol..

[36] Romani P, Valcarcel-Jimenez L, Frezza C, Dupont S (2021). Crosstalk between mechanotransduction and metabolism. Nat. Rev. Mol. Cell Biol..

[37] Davidson PM, Cadot B (2021). Actin on and around the Nucleus. Trends Cell Biol..

[38] Kim J-K (2017). Nuclear lamin A/C harnesses the perinuclear apical actin cables to protect nuclear morphology. Nat. Commun..

[39] Shi, L. *et al.* Self-healing silk fibroin-based hydrogel for bone regeneration: dynamic metal-ligand self-assembly approach. *Adv. Funct. Mater.* **27**, (2017).

[40] Haralick RM, Dinstein I, Shanmugam K (1973). Textural features for image classification. IEEE Trans. Syst. Man Cybern..

