# Supplementary material for: Brain microvasculature endothelial cell orientation on micropatterned hydrogels is affected by glucose level variations
Source: Sci Rep. 2021 Oct 4;11:19608. doi: 10.1038/s41598-021-99136-9 (PMC8490407; doi:10.1038/s41598-021-99136-9)
Supplement: Supplementary file 1 — Supplementary Information. [file 41598_2021_99136_MOESM1_ESM.pdf]

Brain microvasculature endothelial cell orientation on micropatterned hydrogels is affected by glucose level variations

A.M. Porras Hernandez[1], L. Barbe[1], H. Pohlit[1], M. Tenje[1*#], M. Antfolk[2, 3*#]

[1]Dept. of Materials Science and Engineering, Science for Life Laboratory, Uppsala University, Uppsala, Sweden.

[2]Dept. of Biomedical Engineering, Lund University, Lund, Sweden.

[3]Biotech Research and Innovation Centre, University of Copenhagen, Copenhagen, Denmark

[*]maria.antfolk@bme.lth.se, maria.tenje@angstrom.uu.se

[#]M.T and M.A should be considered joint senior author

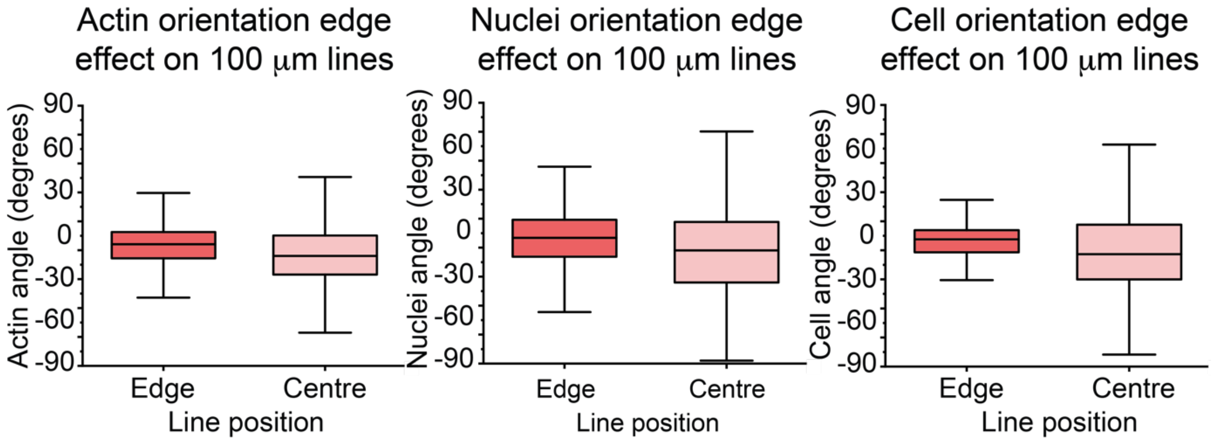

Supplementary Figure S1. Brain microvascular endothelial cells display an edge effect and align more along the edge than in the centre of the 100 µm line. Box plots of actin orientation, nuclei orientation and cell orientation on the edge and centre of the 100 µm line.

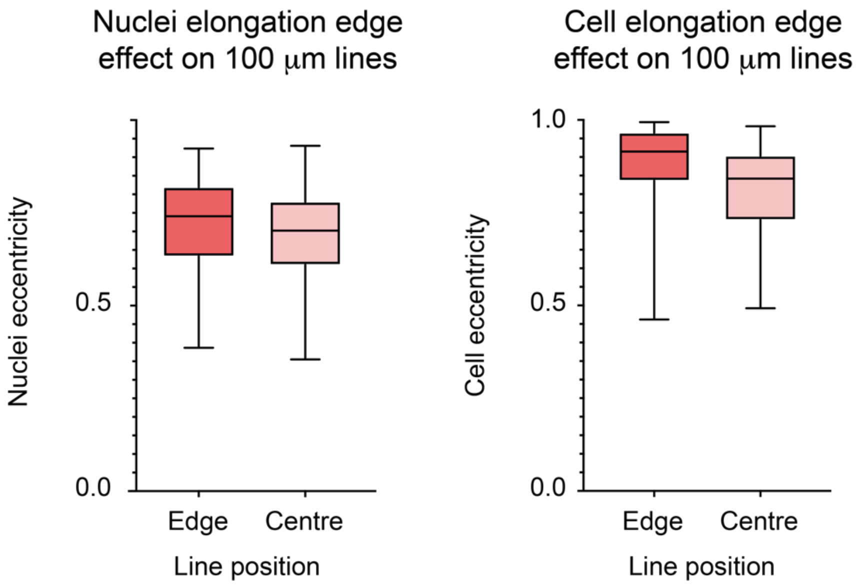

Supplementary Figure S2. Brain microvascular endothelial cells display an edge effect and are more elongated along the edge than in the centre of the 100 µm line. Box plots of nuclei elongation and cell elongation on the edge and centre of the 100 µm line.

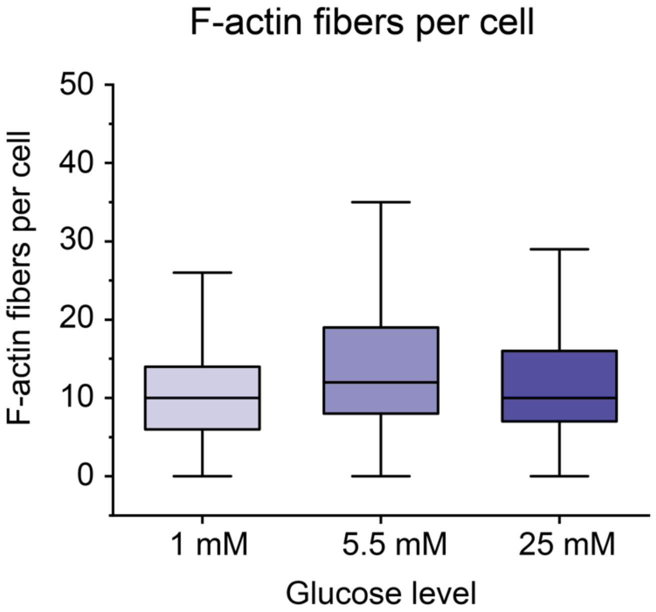

Supplementary Figure S3. Number of F-actin fibers per cell depending on different glucose conditions. Box plot of the number of F-actin fibers per cell under the different glucose conditions low (1 mM), normal (5.5 mM), or high (25 mM).

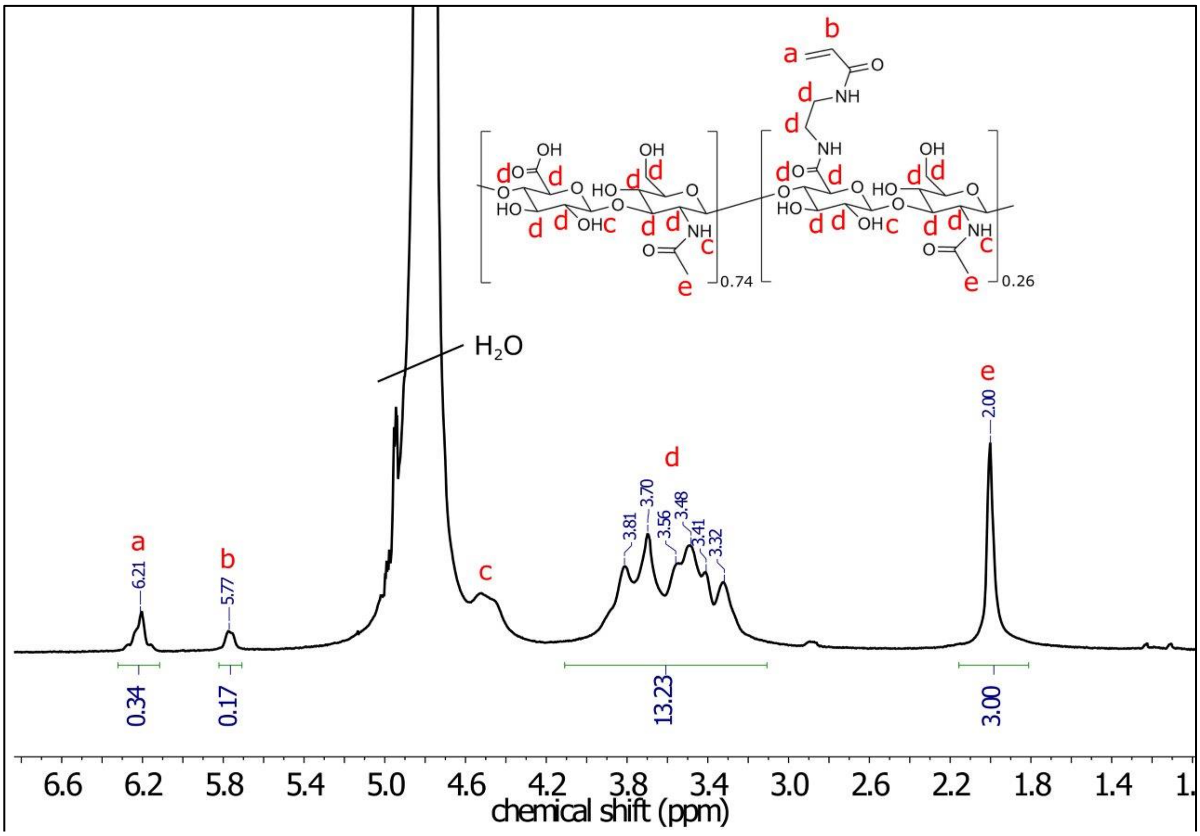

Supplementary Figure S4. ^{1}H NMR spectrum of hyaluronic acid acrylamide (HA-am). The spectrum indicates approximately 14% degree of modification of hyaluronic acid with acrylamide groups.

Supplementary Table S1. Variance analysis of the nuclei orientation of bEnd.3 cells grown on RGD peptide patterns of varying width. An Ansari-Bradley test compensated for multiple comparisons was performed to the data.

Difference of levels	hypothesis	P-value
**10 - 25**	1	<0.0001 ***
**10 - 50**	1	<0.0001 ***
**10 -75**	1	<0.0001 ***
**10 -100**	1	<0.0001 ***
**10 - NP**	1	<0.0001 ***
**20 - 50**	1	<0.0001 ***
**20 -75**	1	<0.0001 ***
**25 -100**	1	<0.0001 ***
**25 - NP**	1	<0.0001 ***
**50 - 75**	0	2.675654
**50 - 100**	0	4.781947
**50 - NP**	1	<0.0001 ***
**75 - 100**	0	13.72066
**75 - NP**	1	<0.0001 ***
**100 - NP**	1	<0.0001 ***

Supplementary Table S2. Variance analysis of the cell orientation of bEnd.3 cells grown on RGD peptide patterns of varying width. An Ansari-Bradley test compensated for multiple comparisons was performed to the data.

Difference of levels	hypothesis	P-value
**10 - 25**	1	<0.0001 ***
**10 - 50**	1	<0.0001 ***
**10 -75**	1	<0.0001 ***
**10 -100**	1	<0.0001 ***
**10 - NP**	1	<0.0001 ***
**25 - 50**	1	0.0295
**25 -75**	1	<0.0001 ***
**25 -100**	1	<0.0001 ***
**25 - NP**	1	<0.0001 ***
**50 - 75**	0	0.26975
**50 - 100**	1	<0.0001 ***
**50 - NP**	1	<0.0001 ***
**75 - 100**	1	0.00386 ***
**75 - NP**	1	<0.0001 ***
**100 - NP**	1	<0.0001 ***

Supplementary Table S3. Variance analysis of the actin orientation of bEnd.3 cells grown on RGD peptide patterns of varying width. An Ansari-Bradley test compensated for multiple comparisons was performed to the data.

Difference of levels	hypothesis	P-value
**10 - 25**	1	<0.0001 ***
**10 - 50**	1	<0.0001 ***
**10 -75**	1	<0.0001 ***
**10 -100**	1	<0.0001 ***
**10 - NP**	1	<0.0001 ***
**25 - 50**	1	<0.0001 ***
**25 -75**	1	<0.0001 ***
**25 -100**	1	<0.0001 ***
**25 - NP**	1	<0.0001 ***
**50 - 75**	0	0.19308
**50 - 100**	1	<0.0001 ***
**50 - NP**	1	<0.0001 ***
**75 - 100**	1	<0.0001 ***
**75 - NP**	1	<0.0001 ***
**100 - NP**	1	<0.0001 ***

Supplementary Table S4. Means analysis of the nuclei elongation (eccentricity) of bEnd.3 cells grown on RGD peptide patterns of varying width. A one-way ANOVA with Tukey post hoc for multiple comparisons was performed to the data.

Difference of Levels	Diference of Means	SE of Diference	95% CI	T-Value	P-Value	
**10 - 25**	-0.03778	0.00985	(-0.06585; -0.00970)	-3.83	0.002	**
**10 - 50**	-0.07685	0.00985	(-0.10493; -0.04878)	-7.8	0.000	***
**10 -75**	-0.09203	0.00985	(-0.12010; -0.06396)	-9.34	0.000	***
**10 -100**	-0.06625	0.00985	(-0.09433; -0.03818)	-6.73	0.000	***
**10 - NP**	-0.14609	0.00985	(-0.17417; -0.11802)	-14.83	0.000	***
**25 - 50**	-0.03908	0.00985	(-0.06715; -0.01100)	-3.97	0.001	***
**25 -75**	-0.05426	0.00985	(-0.08233; -0.02618)	-5.51	0.000	***
**25 -100**	-0.02848	0.00985	(-0.05655; -0.00040)	-2.89	0.044	*
**25 - NP**	-0.10832	0.00985	(-0.13639; -0.08024)	-11	0.000	***
**50 - 75**	-0.01518	0.00985	(-0.04325; 0.01290)	-1.54	0.638	
**50 - 100**	0.0106	0.00985	(-0.01747; 0.03867)	1.08	0.891	
**50 - NP**	-0.06924	0.00985	(-0.09731; -0.04117)	-7.03	0.000	***
**75 - 100**	0.02578	0.00985	(-0.00230; 0.05385)	2.62	0.093	
**75 - NP**	-0.05406	0.00985	(-0.08214; -0.02599)	-5.49	0.000	***
**100 - NP**	-0.07984	0.00985	(-0.10791; -0.05177)	-8.1	0.000	***

Supplementary Table S5. Means analysis of the cell elongation (eccentricity) of bEnd.3 cells exposed to varying levels of glucose A one-way ANOVA with Tukey post hoc for multiple comparisons was performed to the data.

Difference of Levels	Diference of Means	SE of Diference	95% CI	T-Value	P-Value
**10 - 25**	-0.02618	0.00903	(-0.05192; -0.00045)	-2.9	0.043 *
**10 - 50**	-0.05791	0.00903	(-0.08365; -0.03218)	-6.41	0.000 ***
**10 -75**	-0.06843	0.00903	(-0.09416; -0.04269)	-7.58	0.000 ***
**10 -100**	-0.07051	0.00903	(-0.09625; -0.04478)	-7.81	0.000 ***
**10 - NP**	-0.05305	0.00903	(-0.07878; -0.02732)	-5.87	0.000 ***
**25 - 50**	-0.03173	0.00903	(-0.05747; -0.00600)	-3.51	0.006 **
**25 -75**	-0.04224	0.00903	(-0.06798; -0.01651)	-4.68	0.000 ***
**25 -100**	-0.04433	0.00903	(-0.07007; -0.01860)	-4.91	0.000 ***
**25 - NP**	-0.02687	0.00903	(-0.05260; -0.00113)	-2.98	0.035 *
**50 - 75**	-0.01051	0.00903	(-0.03625; 0.01522)	-1.16	0.854
**50 - 100**	-0.0126	0.00903	(-0.03834; 0.01313)	-1.4	0.73
**50 - NP**	0.00486	0.00903	(-0.02087; 0.03060)	0.54	0.995
**75 - 100**	-0.00209	0.00903	(-0.02782; 0.02364)	-0.23	1.000
**75 - NP**	0.01537	0.00903	(-0.01036; 0.04111)	1.7	0.53
**100 - NP**	0.01746	0.00903	(-0.00827; 0.04320)	1.93	0.381

Supplementary Table S6. Variance analysis of the nuclei orientation of bEnd.3 cells grown on 10 µm wide line RGD peptide patterns and exposed to varying levels of glucose. An Ansari-Bradley test compensated for multiple comparisons was performed to the data.

**Difference of levels**	**hypothesis**	**P-value**
1 mM vs 5.5 mM	0,000408	1
1 mM vs 25 mM	0,825088	0
5.5 mM vs 25 mM	0,030544	1

Supplementary Table S7. Variance analysis of the actin orientation of bEnd.3 cells grown on 10 µm wide line RGD peptide patterns and exposed to varying levels of glucose. An Ansari-Bradley test compensated for multiple comparisons was performed to the data.

**Difference of levels**	**hypothesis**	**P-value**
1 mM vs 5.5 mM	0,225071	0
1 mM vs 25 mM	0,006516	1
5.5 mM vs 25 mM	0,000008	1

Supplementary Table S8. Variance analysis of the cell orientation of bEnd.3 cells grown on 10 µm wide line RGD peptide patterns and exposed to varying levels of glucose. An Ansari-Bradley test compensated for multiple comparisons was performed to the data.

**Difference of levels**	**hypothesis**	**P-value**
1 mM vs 5.5 mM	0,000620	1
1 mM vs 25 mM	0,112548	0
5.5 mM vs 25 mM	0,000000	1

Supplementary Table S9. Means analysis of the nuclei elongation (eccentricity) of bEnd.3 cells exposed to varying levels of glucose A one-way ANOVA with Tukey post hoc for multiple comparisons was performed to the data.

**Difference of Levels**	**Diference of Means**	**SE of Diference**	**95% CI**	**T-Value**	**P-Value**
**1 mM – 5.5 mM**	-0.01969	0.005995	(0.03375; -0.005630)	4.645	0.003 **
**1 mM - 25 mM**	0.004777	0.005995	(-0.009282; 0.01884)	1.127	0.705
**5.5 mM - 25 mM**	0.02447	0.005995	(0.01041; -0.03853)	5.772	0.0001 ***

Supplementary Table S10. Means analysis of the cell elongation (eccentricity) of bEnd.3 cells exposed to varying levels of glucose. A one-way ANOVA with Tukey post hoc for multiple comparisons was performed to the data.

**Difference of Levels**	**Diference of Means**	**SE of Diference**	**95% CI**	**T-Value**	**P-Value**
**1 mM – 5.5 mM**	-0.02898	0.005467	(-0.04180; -0.01616)	7.497	<0.0001 ***
**1 mM - 25 mM**	0.004008	0.005467	(-0.008814; 0.01683)	1.037	0.7439
**5.5 mM - 25 mM**	0.03299	0.005467	(0.02017; 0.04581)	8.534	<0.0001 ***
